# Routing with Face Traversal and Auctions Algorithms for Task Allocation in WSRN

**DOI:** 10.3390/s21186149

**Published:** 2021-09-13

**Authors:** Jelena Stanulovic, Nathalie Mitton, Ivan Mezei

**Affiliations:** 1 Faculty of Technical Sciences, University of Novi Sad, Trg Dositeja Obradovića 6, 21000 Novi Sad, Serbia; jstanulovic@gmail.com; 2 Maxlinear Austria Gmbh, Europastrasse 8, 9500 Villach, Austria; 3 Inria Lille, 40 Avenue Halley, 59650 Villeneuve d’Ascq, France; nathalie.mitton@inria.fr

**Keywords:** auctions, face routing, GFG routing, greedy routing, wireless sensor and robot networks

## Abstract

Four new algorithms (RFTA1, RFTA2, GFGF2A, and RFTA2GE) handling the event in wireless sensor and robot networks based on the greedy-face-greedy (GFG) routing extended with auctions are proposed in this paper. In this paper, we assume that all robots are mobile, and after the event is found (reported by sensors), the goal is to allocate the task to the most suitable robot to act upon the event, using either distance or the robots’ remaining energy as metrics. The proposed algorithms consist of two phases. The first phase of algorithms is based on face routing, and we introduced the parameter called search radius (SR) at the end of this first phase. Routing is considered successful if the found robot is inside SR. After that, the second phase, based on auctions, is initiated by the robot found in SR trying to find a more suitable one. In the simulations, network lifetime and communication costs are measured and used for comparison. We compare our algorithms with similar algorithms from the literature (k-SAAP and BFS) used for the task assignment. RFTA2 and RFTA2GE feature up to a seven-times-longer network lifetime with significant communication overhead reduction compared to k-SAAP and BFS. Among our algorithms, RFTA2GE features the best robot energy utilization.

## 1. Introduction

Wireless sensor and robot networks (WSRN) consist of the combination of two types of wireless networks that cooperate: sensor networks and robot networks (sometimes called mobile actuators). A sensor network consists of static nodes with sensing capabilities, while a robot network consists of mobile nodes with actuating and enhanced capabilities. The price and abilities (including energy) of the sensor nodes are much lower than the robot’s. Accordingly, the number of sensors monitoring the area is typically much higher. The sensor communication radius is also much lower. All aim to have low power consumption (battery power supply) to enable a long node lifetime. The number of robots is much lower, and they cannot monitor the complete area without moving a lot. Accordingly, without sensors, they would incur a lot of additional costs (energy needed for motor movement is at least one magnitude higher than communication costs) in energy and response delays.

**Figure 1:**
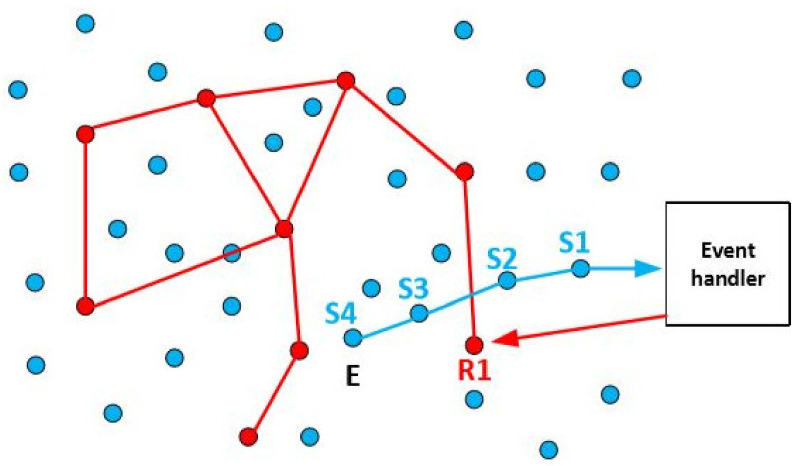
Cooperative WSRN.

A WSRN system, based on these cooperative networks [1], can monitor the area for events using sensors, make a localized decision on which robot should move to the event location, and act upon a detected event(s). A cooperative WSRN network is illustrated in Figure 1 (blue dots are sensors, red dots are robots, blue lines represent the communication lines between the sensors, and red lines, accordingly, are communication lines between the robots). Let us assume an event E occurred near sensor S4, which sensed it and forwarded the information (including the event’s position) to the event handler via sensors S3, S2, and S1. The event handler is an entity that collects the data from sensors and sends the order to robots. The event handler passes the information about the event containing the event location to a particular robot called the collecting robot (in this scenario R1). The event handler chooses the robot to which it randomly sends the information by attaching its ID to the message with the event location. The chosen robot relays the message through the robot network towards the robots in the event vicinity where local robots need to decide which one is the best to act. An alternative way to find the location of a suitable robot is to apply one of the service discovery protocols. However, this centralized solution would have high time and communication costs and is unsuitable for our scenarios. In some scenarios, the monitored event requires only additional information without any action (e.g., video surveillance of the event location). In other applications, such as intelligent indoor monitoring of buildings (e.g., temperature, smoke density, air humidity, vibrations, etc.), upon the detection of the problems (e.g., smoke is detected in some areas of the building), a robot is expected to react immediately and resolve the issue. Robots are beneficial in scenarios where human participation is dangerous or not possible (e.g., rescue activities after natural disasters [2]).

One of the main problems in the wireless sensor and robot networks is how and to which robot(s) to allocate the task. In this paper, it is assumed that the sensors received information about an event that happened somewhere in the network, and the best robot is supposed to react. Accordingly, the task assignment problem (also known as the task allocation problem) is to find the most suitable robot to respond to the event. This problem is in robotic literature well known as a multirobot task allocation problem, and in [3,4] and [5], are reviewed and presented the existing solutions and their variations in WSRN.

In this paper, we tackle the task allocation problem. We proposed four new solutions based on greedy face traversal routing extended with auctions. These new routing algorithms rely on the extension of the GFG routing [6] proposed in our previous work [7]. The motivation for the new algorithms came from earlier work [7] that showed the need to improve the GFGF algorithms in terms of higher efficiency, lower communication costs, and longer network lifetime. The basic scenario on which this paper focuses is the following. The sensors identify the event location, and the location information is sent to one of the robots (robot R1 in Figure 1). The event is somewhere in the area monitored by the robots, but these later need to move to the exact location to perform the task. We use the GFGF2 algorithm to find a robot near the event location in the search radius SR, as in [7], and extend it with auctions to discover whether there is a better robot that can perform the task compared to the one found within the search radius SR around the event. While in [7], the focus is on finding the closest robot to react to the task, here we aim to find the most suitable robot to react based on the metrics that take into account the remaining energy, the distance to the event, and the speed of robot movement.

Auctions used in this work are based on k-SAP auction protocol [8]. The first proposed algorithm uses distance metrics and is called RFTA1. The other proposed algorithms, RFTA2, GFGF2A, and RFTA2GE, use the remaining robot energy as the metrics. Here, we measure the network’s lifetime, energy balancing among robots, and robot utilization.

The main contributions of this paper are the following:

- We improve the existing greedy-face-greedy–based routing solution and apply it to the robot task allocation problem. It results in a more extended network lifetime and better energy balancing using a combination of face traversal routing and auctions based on different metrics evaluated for different scenarios and network topologies.
- We introduce the RFTA2 algorithm, which shows a network lifetime of up to seven times longer than k-SAAP [9] and BFS [10] algorithms with significantly fewer communication costs. It also offers a network lifetime of up to five times longer than the GFGF2A algorithm (for few additional communication costs).
- We introduce RFTA2GE, which features the best robot energy utilization and energy balancing among all robots for additional communication costs. Since the communication costs are usually by an order of magnitude lower than robot movement costs, this is a highly beneficial contribution of RFTA2GE. It features a network lifetime up to seven times longer than k-SAAP [9] and BFS [10] for three times fewer communication costs.
- Within the algorithms RFTA2 and RFTA2GE, we introduce the parameter SR (search radius). It is the radius of the circle which defines the space around the event where the search for the robot is performed. We also determine its optimal value and prove it mathematically.

The rest of the paper is organized as follows. The overview and explanation of the related work protocols are given in Section 2. In Section 3, the system model is presented, and the newly proposed algorithms are presented in the following section. In Section 5 are presented simulation results and a discussion of the obtained results. Section 6 concludes the paper and provides future work proposals.

## 2. Related Work

Here, we present related work on the geographic routing protocols that are the basis for our work presented in this paper. They are face routing [11], greedy routing [12], greedy-face-greedy (GFG) routing [6], greedy-face-greedy-find (GFGF) [7], and auctions [8]. We also review the literature related to the task allocation problem.

### 2.1. Task Allocation

In the multi-robot systems literature, task allocation is a well-studied topic (e.g., the survey [13]). The task allocation strategies can be classified according to their applications [14]. Optimization- and auction-based techniques are described as the most important task allocation strategies [4,5,15].

Task allocation aims to match appropriate robots and specific jobs required by the system most efficiently [16]. The auction-based task allocation approach is one of the possible solutions whereby using auctions, robots bid on a task. The bids are based on certain metrics, and the cheapest one is assigned to the task [17]. Another approach presented in [18] is the greedy approach implemented using the closest robot. In our paper, we are using a combination of these two approaches.

Additionally, another feature to consider is the network’s connectivity with the task allocation problem, which is studied in [19]. The authors in [20] investigate the task assignment problem when a limited communication range constrains robots. They propose both centralized as well as decentralized solutions. In contrast to many task allocation approaches that assign one task at a time per robot, in [21], single robots are performing multiple tasks simultaneously with different priorities.

The geographical routing in the WSRN has become an important research topic. In location-based routing, since the decisions are made based only on location information, there is no need for complex computation. As location-based protocols do not use information about the entire topology but only location information, they are very efficient in terms of routing. A survey and taxonomy of location-based routing for WSN are presented in [22]. They categorized the recent research works into several directions.

To improve the efficiency and lifetime of the network, auctions are used in [9]. They use the localized auction aggregation protocol (k-SAAP). It is shown that the proposed algorithm in some scenarios guarantees 100% efficiency in finding the closest actuator.

RODAA was presented in [10], and the goal of the paper was to optimize robot resources and task completion time. It is based on BFS algorithm [23]. The BFS tree is used to overcome the weakness of the auction algorithm, in which only the single-hop neighbors of the auctioneer can participate in the auction process if the communication range of the robot is limited. Since the tasks must be allocated in a dynamic environment where the robots are mobile, and the tasks occur anywhere at any time, the BFS tree-based approach guarantees conflict-free task allocation.

In Reference [24], an adaptive auction protocol AAP for task assignment in multi-hop wireless actuator networks is proposed, and the scenario that is considered is where all actuators are static, and each of them can obtain the target information from sensors. Unlike existing methods that neglect the adaptive auction area required by dynamic networks, the proposed method uses an adaptive factor that is deduced based on the relation between network characteristics and protocol performance. The robot is considered to be static, so this algorithm is not suitable for comparison with our algorithms.

### 2.2. Face Routing

This type of routing assumes that the network is divided into faces. Face routing protocol [11] forwards the message along with the adjacent faces. Those faces are all intersecting the line connecting source and destination sd. When the message reaches the edge, which is intersecting the sd line, the face is changed. Only intersections that are closer to the destination, compared to the last intersection point, are considered. There are several variations of the algorithm called ‘before crossing’, ‘after crossing’, and ‘the left-hand’ and ‘right-hand’ rule. The left-hand rule means that the message is traversed along the edge placed counterclockwise from the previously examined edge. The right-hand rule assumes that the message travels along the edge, lying in a clockwise direction.

The before/after crossing variants are used for the next face selection. Using the before-crossing variant, the message is forwarded along the edge, not intersected by the line connecting source and destination. In the other variant, the other edge is selected, i.e., the one intersected by the line sd. The third possibility is called ‘the best angle variant’ minimizing the angle between the possible edges to choose.

### 2.3. Greedy Routing

Greedy routing [12] is one of the simplest protocols for geographical message forwarding. Using this protocol, the message is continually forwarded closer to the destination. The message routing is started from a source node (e.g., robot) towards the destination node at a specific location. To decide where to forward the message, the sender checks which neighbors are closer to the destination than itself. Accordingly, the next node is the new source performing the same action to forward the message further. This operation continues until the destination is reached or none of the neighbors are closer to the destination than the current source. In such a case, it is impossible to forward the packet; routing fails, and the packet is dropped. This is the main drawback of greedy routing. There are several recovery techniques and protocols (e.g., GFG).

### 2.4. Greedy-Face-Greedy (GFG) Routing

Greedy-face-greedy routing (GFG) [6] is based on the combination of greedy and face routing. In the greedy phase, the packet is forwarded along with the nodes in such a way that the next node is the closest of all potential neighboring nodes to the destination compared with the current node. When there are no closer neighbors, the face phase of the algorithm is started, and it is performed until the closer node is found. The main idea is that the message is forwarded to the next node on the face until the intersection between the line connecting the source and destination node and the line connecting the current and next node is found. If such an intersection is encountered, the face is changed, and the message is forwarded to the node in the next face. The GFG algorithm switches between the greedy and face phase as many times as needed until the delivery is performed since this algorithm is routing with guaranteed delivery.

### 2.5. Greedy-Face-Greedy-Find (GFGF) Algorithms

**Figure 2:**
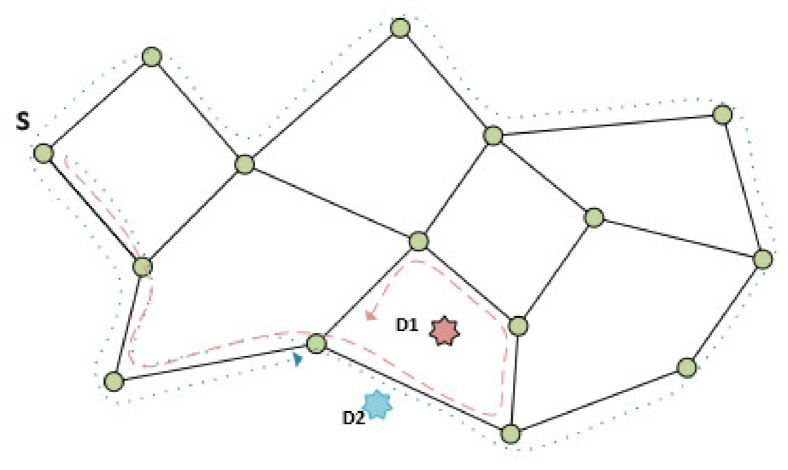
Two scenarios: D1 in and D2 outside of the network area.

Greedy-face-greedy-find (GFGF) algorithms are based on GFG when it is applied to the scenario where destination D (event location) is not part of the network [7]. There are two algorithms designated as GFGF1 and GFGF2. The main issue is the definition of the stop criteria since the destination is not part of the network and cannot be reached with the standard GFG, and accordingly, the routing would never finish, and the event would never be found. GFGF1 routing stops when the same edge of a face has already been visited. In that way, with this algorithm, the event is surrounded by the nodes in the face around it (e.g., event D1 in Figure 2). Additionally, it is shown that the node closest to D could always be found.

Furthermore, in the case where the event is somewhere around the outer face of the network (e.g., event D2 in Figure 2), GFGF1 routing has high communication costs since the face around the event is the outer face. GFGF2 algorithm is used to cope with this problem when the event is outside of the network. The difference to the GFGF1 is the stop criteria.

**Figure 3:**
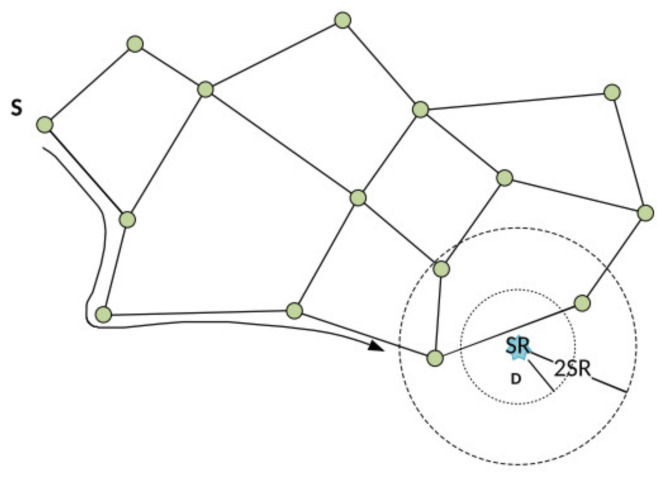
Illustration of the GFGF2 algorithm.

Both possible scenarios for the GFGF2 are illustrated in Figure 3. If the robot could not be found within the search radius SR, the routing would continue all around the network (following the so-called outer face). The routing stops when it comes back to the node where it started, even though it is not inside the search radius SR around the event. In the search scenario with search radius 2SR, there is a robot inside, and the stop criterion is satisfied with less communication overhead. The closest robot is found in 55–90% of routings, compared to GFGF1, where it is almost 100% [7].

### 2.6. Auctions

The market-based approach and auctions are often used in robotics to do task allocation [13]. However, the complete network communication graph (i.e., each robot can communicate with each other) is often assumed, which is a hard constraint. Instead of flooding the whole network, localized multi-hop auctions called k-SAP are introduced in [8]. The auctions are organized by a robot called auctioneer, which sends a message to all of his first neighbors. The ones which, according to the used metric, can do the task bid back. The bids are searched only among the robots which are located within k-hops from the auctioneer. Then the auctioneer decides which robot is the best to run the task. The example of a metric based on multi-objective goals is given in [25]. However, most studies assume that every target location is reachable for the robots, which does not apply to the scenarios in which obstacles may render some locations [26].

In [26] and [27], the authors investigate the task assignment problem with the following assumptions: (1) there are multiple targets with specific requirements, (2) the robots have different capabilities, and (3) for some tasks, there is a need that different robots with different capabilities visit some locations. Reference [26] implies a complete graph where all the robots have information about all the target locations. During the auctions process, each robot bids for each task. In [27], the authors investigate a similar problem but with a limited communication range. The subnetworks are created locally and the auctions are organized by the local leader robot. They propose a decentralized auction algorithm that first merges local information of a robot subnetwork and then applies a marginal-cost-based strategy to resolve the task assignment problem.

## 3. System Model

We assume that the network consists of two types of nodes: static sensors and mobile robots. Both sensors and robot nodes are considered to be aware of their position. The sensors obtain information about the event’s location, and a robot is supposed to be assigned to react to that event. All the robots in the network have the same transmission radius *r*, which means that each robot can communicate only with its first robot neighbors located inside the radius *r*. Accordingly, the robot network is connected all the time but does not form a complete communication graph (i.e., not all robots can directly communicate with each other) since robot communication is modeled as a unit disc graph (UDG). Communications among robots are done in a multi-hop manner. The communication between every two robots is possible by sending the communication messages over other robots, which only relay messages towards the destination. We assume that robots and sensors cannot communicate.

We assume that all of the robots know their position (e.g., by having localization hardware on itself). The collecting robot is the one that gets the information about the event location and informs the other robots about the event. The typical solution of the task allocation problem is centralized, assuming a complete communication graph, which can be optimal but needs complex computations (e.g., by using linear integer programming [28]). In this paper, we propose several localized solutions based on a multi-hop communication model. In such a model, the collecting robot has the information about the event obtained from sensors via the event handler. The collecting robot is considered the source, and it starts the routing towards the other robots (e.g., those closer to the event). The message containing the location of the event is then forwarded through the robot part of the network. We consider two scenarios in terms of network topology. The first is with uniform random placement of the robots, and the second is uniform random placement with a hole around the center of the monitored area. To perform the message routing and eventually allocate the robot for the task on the event destination, we focus on the algorithms based on greedy face traversal routing combined with auctions.

To reduce the complexity of routing algorithms, only some of the available communication links are considered. To do so, we need to apply a graph reduction tool that maintains connectivity. Compared to the others, the unit disc graph (UDG) has no restrictions other than a fixed transmission radius, but, on the other hand, has a high interference due to the high number of connections. The strongest restriction that does not affect and even preserves the connectivity is based on a spanning tree. However, it features a low diversity of routing paths and therefore has less possibility for energy balancing and routing optimizations. Another solution that has many favorable features is the Gabriel graph (GG). The latter has a significantly lower number of connections, which reduces communication overhead, and collisions compared to UDG. In addition, it induces a planar graph, contains the Euclidian minimum spanning tree as a subgraph, and has an optimal energy spanner [29]. On the other hand, the relative neighborhood graph (RNG) removes more edges and has lower diversity in paths than GG. The degree of the graphs (average number of the neighbors) is in the following relations:

d(UDG) > d(GG) > d(RNG)

Accordingly, there are fewer routing paths for the RNG compared to GG. On the other hand, GG features a lower degree than UDG, accordingly having fewer communication links than UDG. For those reasons, we use the Gabriel graph of random UDG as a middle solution for the number of connections compared to UDG and RNG to model the robot network in this paper.

**Definition** **1.** *Task allocation problem.*For a given:
*S = {S1, S2, … Sm}*, set *S* of *m* sensors,
*R = {R1, R2, …Rn}*, set *R* of *n* robots with known locations,
*E = {E1, E2, …En}*, initial energies of robots where *E1 = E2 … = En*, and
*T = {T1, T2, …Tk}* set of *k* successive events on known target locations detected by sensors; the problem is to find the best robot to react upon the event in each round and to assign the task for all consecutive events. Only a single-task-single-robot assignment is possible during one round. Accordingly, the problem is to pair the robots and the tasks with the minimum total cost. The cost could be a function of distance, energy, communication costs, time, or some combination of parameters. Here, we want to maximize the network lifetime by minimizing each robot’s energy spent to do the task. The task allocation problem can be mathematically formulated as:
Find the assignment matrix *A* = {*a_ij_*} where *i = 1, 2, … n* and *j = 1, 2, … k*
and
$$min\underset{ij}{\sum }a_{ij\;}E_{ij}$$
$E_{ij}$—energy needed for performing the task *j* with conditions
$$\overset{n}{\underset{i=1}{\sum }}a_{ij}=1,\;\forall j\;\overset{k}{\underset{j=1}{\sum }}a_{ij}=1,\;\forall i$$
where $a_{ij}=1$ if the robot *j* is matched to the task *i*; otherwise, $a_{ij}=0$. The first condition implies that one task cannot be assigned to more than one robot and the second one that there cannot be assigned more than one task to a single robot.
The total energy consumption for the robot *i* to perform a task *j* must be smaller than the initial energy of robot $(R_{i}$):$$\overset{k}{\underset{j=1}{\sum }}E_{ij}a_{ij}\leq E\left(R_{i}\right)$$
The energy needed to perform the task is considered to be dependent on distance to the task location $d_{ij}$ and robot’s speed v:$$E_{ij}=f\left(v,d_{ij}\right)$$

The previous mathematical formulation of the task assignment problem is hard to be solved. It belongs to the task assignment problem with side constraints [30] which is NP-hard. Although it is possible to use a heuristic to solve this optimization problem (e.g., Hungarian algorithm [31]), this centralized formulation assumes that all events are known in advance, which is a very hard assumption for real applications.

Here is the list of assumptions to our localized algorithms given in the next section:

- All the robots in the network have the same transmission radius r, and each robot can communicate only with its first neighbors located inside of the radius r.
- All the robots know their position, and the position of the current event is known. Other events are not known *a priori*.
- All the robots have a certain amount of energy at the beginning of the routing process called ‘initial energy’. In the beginning, we assume it is at 100%. While performing the task, the robot energy is drained proportionally to the distance traveled.
- The search radius SR is a circle around the event where the robot is searched; the routing stops if any robot is identified. Since the robots can determine both their position and the event’s position, they can decide locally whether they are in the circle or not.
- During auctions, robots calculate the energy needed to do the task. If it does not have enough energy to do the task, the robot does not bid back.
- To evaluate the algorithms, we focus on communication costs and the network lifetime.
- Communication costs are based on the number of messages needed to route the message from the source to the destination during one round of simulations (i.e., for one event).
- The network is considered alive until a task cannot be allocated because none of the neighboring robots have enough energy to perform it, and no one bid.
- The network is assumed to be connected. One particular strategy for it could be finding the (minimal) connected dominating set. Communications would be used to update and maintain connectivity. Maintaining the network’s connectivity is out of the scope of this paper. It remains for future work.

## 4. RFTA Algorithms

We propose a family of four new algorithms, designated as RFTA (Routing with Face Traversal and Auctions), to further improve already suggested GFGF routing algorithms. The basic idea is to improve their efficiency using auctions. Four new algorithms are described in the following subsections. All of them are two-phase algorithms. First, greedy-face-greedy-find (GFGF) routing is active until one of the stop criteria is reached. It is followed by the second phase—the phase with auctions. In the auctions phase, the last robot who got the message with the GFGF algorithm organizes the auctions (i.e., this robot is the auctioneer). Auctions are always performed in the way that the auctioneer sends one message to all of its closest neighbors (i.e., 1-hop neighbors). Neighbors bid according to the criteria based on the distance to the event (RFTA1) or the robot’s remaining energy (RFTA2 and RFTA2GE). In the latter case, each robot calculates the bid according to the remaining energy it would have left after doing the task.

### 4.1. RFTA1 Algorithm

The RFTA1 algorithm consists of two phases. In the first phase, the routing is done using the GFGF2 algorithm. The auctioneer sends the message about the event to all of its neighbors, as well as its bid, which is assumed to be the best at the beginning of the auctions. To be communicationally efficient (i.e., low number of messages), only the robots closer to the event destination D (task) than the auctioneer send their bids. The auctioneer collects all the offers and decides which robot is the best to run the task, based on the distance to the event location D. Pseudocode for RFTA1 is given in Algorithm 1.
**Algorithm 1.** Pseudocode for RFTA1 algorithm**Do** GFGF2 **until** a robot within the search radius SR around the event destination D is found **or until** the next node has already been examined (in that case, take the last examined robot as the winner)**Assign** that robot to be the auctioneerAuctioneer: Start auctions
The auctioneer sends the message to all its first neighbors, calling for the closest to the event destination DRobots bid back if they are closer to the eventThe auctioneer decides which is the best to respond and allocate the task

### 4.2. RFTA2 Algorithm

In this algorithm, the remaining energy of the robot’s battery is used as the metric. The first approximation for the energy consumed for the robot movement is based on the ‘linear rule’ [32], where the energy consumption is calculated as a linear function of the distance. In this paper, each of the neighbors’ bids is based on the following calculation, which takes into account the remaining energy they have, their distance (*d*) to the event location D, and their speed (*vRobot*), using the Equation (1) taken from [33],

(1) $$EnergyLoss=6.25\cdot vRobot\cdot d+9.79\;\cdot d+3.66\;\cdot \frac{d}{vRobot}$$

A robot calculates the remaining energy in case it is considered to be allocated for the task (distance d is sent as an auction parameter by the auctioneer). The robot bids only if it estimates to have enough energy to run the task. The auctioneer decides which robot is the most suitable for the task.

There are four possible cases to make the decision and allocate the task to one of the robots. First, if more than one of the robots bid for the task, the auctioneer chooses the one with the most remaining energy left after finishing the task. In the second case, if only one robot bids for the task, it is assigned the task. The third case assumes that if none of the robots bid due to low remaining energy, they cannot perform the task. In that case, the auctioneer assigns the task to itself (provided enough energy). The last, fourth case would be when no robot bids for the task and the auctioneer robot does not have enough energy to perform it. It is recognized as the stop criterion for the RFTA2 algorithm and, consequently, network lifetime is considered exhausted.

#### Update of the Network

Once a robot is assigned a task, it moves to the task coordinates (i.e., event destination D), and the network is updated. The update means that the neighbor robots are recalculated to fulfill the communication radius criteria and Gabriel’s graph.

In this way, the routing can be done in rounds as long as there is a robot to react, or all robots have enough remaining energy. With this criterion, the algorithm stops if in the current round, there is no robot to react.

**Figure 4:**
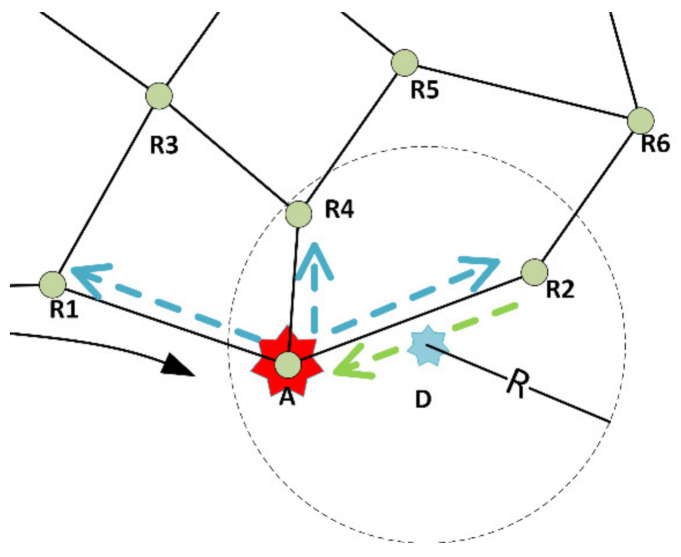
Illustration of the RFTA2 routing.

Figure 4 shows the part of the network where a node inside the radius SR is reached with the first part of the RFTA2 algorithm. This node is assigned to be the auctioneer A. It sends messages to its first neighbors (R1, R4, and R2) illustrated with blue arrows. Node R2 is bidding to perform the task; its message is sent back to the auctioneer and illustrated with a green arrow. Pseudocode for RFTA2 is given in Algorithm 2.
**Algorithm 2.** Pseudocode for RFTA2 algorithm**Do** GFGF2 **until** a robot within the search radius SR around the event destination D is found or **until** the next node has already been examined (in that case, assign the last examined robot as the winner)**Assign** that robot to be the auctioneerStart auctions
The auctioneer sends the message to all of its first neighbors, asking for bids to perform the task at the event destination DRobots calculate their remaining energy and bid accordingly
The auctioneer decides to which robot to assign the task based on the bids
**If** #bids > 1
◦The auctioneer assigns the task to the one with the most remaining energy after completing the task
**Else If** #bids==1
◦The single bidding robot is allocated the task
Else
◦**If** the auctioneer has enough energy, it takes the task, **otherwise**, it stops

The robot that has been assigned the task moves to the coordinates of the task and the network is updatedStart a new round, **go to** 1, and **repeat** steps 1–5, stop when there is no robot to react due to the low remaining energy

### 4.3. GFGF2A Algorithm

GFGF2A is the extension of the GFGF2 algorithm, developed to compare the new RFTA and GFGF algorithms fairly. The extension assumes that all the robots start the routing sequence with the same energy level (assumed to be at 100%). When the algorithm stops because one robot in the radius SR around the event destination D is found, that robot moves to the coordinates of D to perform the task (Algorithm 3). The energy that the robot consumes to perform the task is calculated based on Equation (1).
**Algorithm 3.** Pseudocode for GFGF2A algorithm**Do** greedy **until** delivery or failure**If** failure **then**
Search the next node on the face, based on the right-hand rule**If** (node is within the radius SR around the event destination D)
◦finish ends

**Repeat** steps 1 and 2 **until** the node within the search radius SR is found or **until** the next node has already been examinedThe last robot reacts, and its energy is consumed to perform the task on destination D is calculatedThe robot which is assigned the task moves to the coordinates of D, and the network and its neighbors are updatedStart new round, **repeat** steps 1–5**Stop** when there is no robot to react due to the low remaining energy

### 4.4. RFTA2GE Algorithm

To improve RFTA2, we propose the greedy extension of this algorithm designated as the RFTA2GE. It is based on the method proposed in [34]. After the reacting robot is chosen, its first neighbors are examined to determine whether some of them have energy left after potentially running the task in the destination D. Auctions are started as in RFTA2, and the reacting robot chooses some of the neighboring ones to react. Its first neighbors are examined as in RFTA2, but also their first neighbors are informed about the event, and they bid as well if they can offer to do that task on the coordinates of the destination D. It is given in the Algorithm 4 by pseudocode.

The idea is that this greedy extension improves the performance of the RFTA2 in terms of longer network lifetime at the cost of extra communication.
**Algorithm 4.** Pseudocode for RFTA2GE algorithm**Do** GFGF2 **until** a robot within the search radius SR around the event destination D is found or **until** the next node has already been examined (in that case, assign the last examined robot as the winner)Assign that robot as the auctioneerStart auctions
The auctioneer sends a message to all its neighbors to bid for the task in destination DRobots check whether they have enough energy for that and bid backEach of the neighbors send the message to all their first neighbors and bid back in case they have enough energy to do the task
The auctioneer selects the robot to react based on the bids:
**If** #bids > 1
◦The one with the most remaining energy after doing the task is chosen.
**Else If** #bids==1
◦The single bidding robot is allocated the task
Else
◦**If** the auctioneer has enough energy, it takes the task; **otherwise**, it stops; **go to** 7

The selected robot moves to the coordinates of the task and the network is updatedThe new round is started, **repeat** the steps 1–5The algorithm ends when the robot to react does not have enough energy to run the task

**Figure 5:**
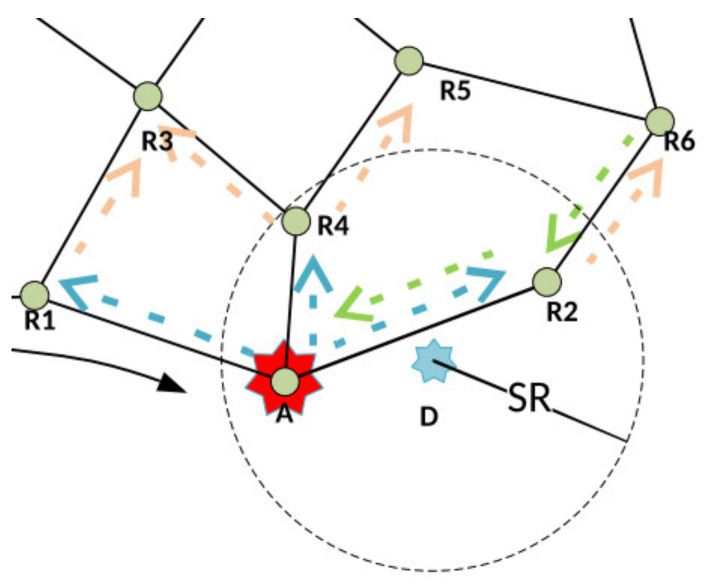
Illustration of the RFTA2GE routing.

Figure 5 shows part of the network where using the first part of RFTA2GE algorithm reaches a node inside the radius SR. It is assigned as the auctioneer A. It sends messages to its first neighbors R1, R4, and R2 (blue arrows). The greedy extension is depicted with orange arrows where messages are also sent to the second neighbors (R3, R5, and R6 in Figure 5). Green arrows show one node that bids back to perform the task at location D. In this example, that is node R6, and it is sending a message to R2, which further informs the auctioneer.

### 4.5. Optimal Radius Value for RFTA2 and RFTA2GE Algorithms

In RFTA2 and RFTA2GE, the search radius SR will be changed from SR to 4SR in SR steps (where SR = 0.1). It can be shown that the best results in terms of network lifetime are obtained for the value of 2SR. Here, we present Lemma 1 and Lemma 2 to prove that.

**Lemma** **1.** *The lower bound of the probability P(R) of finding at least one random point within a circle with the radius R (around one random point) and center that is within the unit square is given by (2)*

*P(R)* ≥ 1 − (1 − π*R^2^/4)^*N*−1^ (2)

*where N is a sufficiently high number of random points within the unit square.*

**Proof** **of** **Lemma** **1.** Using geometrical probability, the worst case of finding one random point out of *N* random points within a circle with the radius R in the unit square is the case when the random point is in the unit square corner. If we express the probability P_out_ of all other *N* − 1 points that are outside of this circle in the corner, then *P(R)* ≥ 1 − *P_out_*^*N*−1^, where *P_out_* = 1 − π*R^2^/4 is the probability that one point is outside of this circle and π*R^2^/4 is the probability of one point that is inside the quarter circle centered in the unit square corner. □

**Lemma** **2.** *For the set of N random points in unit square and the set of search radius {SR, 2SR, 3SR, and 4SR; SR = 0,1}, the best value (i.e., incurring the highest network lifetime) for the search radius used in RFTA2 and RFTA2GE algorithm is 2SR*.

**Proof** **of** **Lemma** **2.** For a sufficiently high number of points, e.g., *N* = 100, according to Equation (2), *P*(0,2) = 0,96 means that the probability of finding at least one robot is more than 95%, and accordingly for 2SR case, the best results are to be expected. To prove it, we show that the other three cases (SR, 3SR, and 4SR) feature worse results. According to Equation (2), *P*(*SR* = 0,1) = 0.54; thus, finding at least one robot within a circle with a search radius SR = 0,1 is not probable in almost half of the cases, and routing fails in those cases. As it holds that *P*(3*SR* = 0,3) = *P*(4*SR* = 0,4) = ~1, at least one robot is (almost) always found. However, due to the larger search radius, the probability that more than one robot is found increases accordingly. Since most of the routings start from the outside of the circle around the event, it encounters the first robot within a circle positioned closer to the circumference than to the center of the circle (event location). This can also be confirmed by the fact that the probability of having a random point in the outer half of the circle area (probability is 1−1/4) is three times higher than within the central half of the circle area (probability is *π*(*SR*/2)2/*πSR*2 = 1/4). Accordingly, the routing stop criterion is met earlier, and the robot is at a larger distance to the event, accordingly spending more energy to come to the event location and thus reducing the network lifetime. □

### 4.6. Complexity Analysis

To analyze the complexity of our algorithm, we are proposing two Lemmas.

**Lemma** **3** **(RFTA2** **upper** **bound).** The number of routing steps for RFTA2 and RFTA2GE is upper bounded by O (n*c), where n is the number of robots and c is the number of edges representing communication links.

**Proof** **of** **Lemma** **3.** In [11], it is shown that GFG is upper bounded by O (n*c) provided that the left-/right-hand rule is not changed while exploring faces, a message explores a face at most once, and then the algorithm stops, or message is advanced to another face, and all visited faces are mutually distinct. This assumption holds for our face traversal strategy. Auctions, as well as greedy extension, are in *O (1)* since it requires a constant number of rounds: three communication rounds (1) round for information, one round for bidding, and one round for the task allocation, and six routing steps (2) rounds for information, two rounds for bidding, and two rounds for the task allocation, respectively. Accordingly, RFTA2 and RFTA2GE are upper bounded by O (n*c). □

**Lemma** **4** **(communication** **complexity** **upper** **bound).** *RFTA2 communication complexity is upper bounded by O (n^2^ + n).*

**Proof** **of** **Lemma** **4.** Let M—number of messages for the task allocation

*F*—number of internal faces of the graph

*deg*—average number of neighboring nodes, deg ~ (n−1)*(πR^2^/A) where *A* is area being monitored

From [35] *F = c − n + 1*, and from planar graph theory, it is well known that

*n−1 < c < 3n*

M = (F + 1)*deg + 1 + 1 = (c – n + 2)*deg + 2

O(M) = O (c – n + 2)*O (n) = O (c*n − n*n + 2n) = O(n^2^ + n)

Accordingly, the communication complexity of *M* is upper bounded by *O (n^2^ + n)*. □

The comparison of ours and the algorithms from literature based on communication complexity, robot mobility, method, and simulation environment is given in Table 1.

## 5. Results and Discussion

We assume that an event is detected by one of the sensors (having event location information) in all algorithms. Event location information is routed to the event handler (see Figure 1), which sends it to one of the robots. Within the robot network, the most suitable one for the task is allocated using our algorithms. The simulation scenarios are written using the C programming language. Network parameters used in simulations for all algorithms are as follows. Random and random with connected hole network topologies based on random Gabriel graph of random UDG are used. In both topologies, the nodes are uniformly distributed in the field. The monitored area is a unit square, the node coordinates are between 0 and 1, the network consists of 100 nodes (robots), and the communication radius r is varied from 0.15 to 0.55 in steps of 0.05. According to [37], there is a sharp edge for *r < ((ln n)/πn)*^1/2^ = *r_C_* ~ 0.12 below which the network is not connected (which is also confirmed by our simulations). Hence, we set a lower bound for the range to 0.15. For values higher than 0.55, the network becomes too dense, approaching a complete graph. Each round of simulations is repeated 100 times. Results are presented as averaged values of 100 or 10,000 simulations. In the graphs with 10,000 simulations, due to the length of one simulation set and conclusion obtained from other simulations, that for *r* > 0.35, both communication costs and network lifetime are becoming constant, we changed the range of r to 0.15–0.35. In the graphs, we show the simulation results together with their 95% confidence intervals.

All distance-based parameters are measured in meters, robot velocity (v) in m/s, communication costs in the number of messages, network lifetime in the number of rounds, and remaining energy as a percentage.

**Figure 6:**
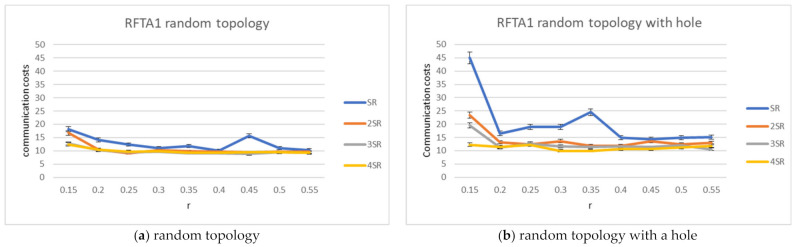
Communication costs for the RFTA1 algorithm.

**Figure 7:**
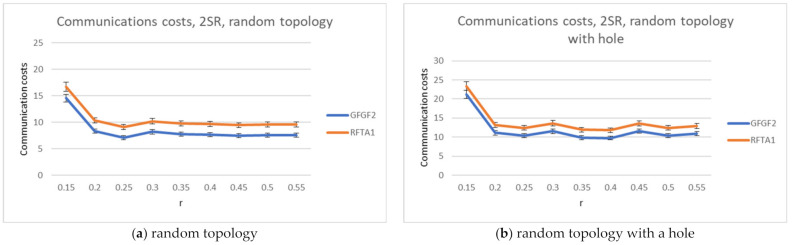
Communication costs comparison for the RFTA1 and GFGF2 algorithms.

**Figure 8:**
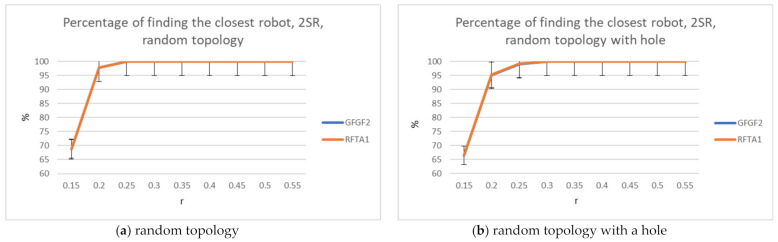
Percentage of finding the closest robot, comparison for the RFTA1 and GFGF2 algorithms.

For the RFTA1 algorithm, the average communication costs and percentage of finding the closest robot to the task were measured and compared to GFGF2. The results are shown in Figure 6, Figure 7 and Figure 8. The results show that RFTA1 has higher communication costs in terms of messages than GFGF2, with almost the same success rate of finding the closest robot for the task. It seems that introducing auctions does not bring any benefit for both topologies.

For the RFTA2 algorithm, the measured values were communication costs, in terms of the number of messages, the lifetime of the network, measured in rounds, and the remaining energies of the robots after the algorithm stop criterion is met. The stop criterion is met when none of the robots participating in the auctions have enough energy to do the task.

Simulations are executed in rounds. In each simulation round, one event occurs at random coordinates, which are not part of the network. The speed of the robots is assumed to be a constant v = 0.76 m/s, as in [33]. For distance calculation, the coordinates of the nodes were multiplied by 10 and assumed in meters.

**Figure 9:**
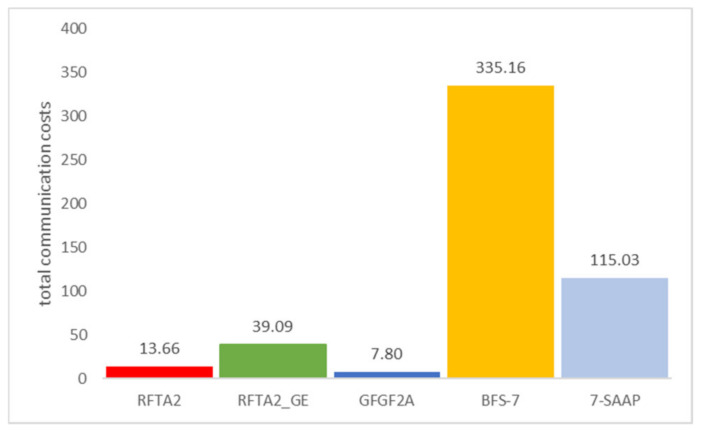
Total communication costs (for 2SR and r = 0.2).

The algorithms proposed in this paper are compared with our algorithms previously presented in [7] as well as with two algorithms from literature k-SAAP [37] and BFS [9]. For both algorithms, the bid metrics are based on the energy and the distance (see Equation (1), [33]). In that way, all algorithms are using the same bidding metrics for a fair comparison. Values of k in k-SAAP and hopmax in BFS are chosen based on the thorough simulations. For k-SAAP, it is 7. For lower values, the network lifetime is very low. The reason is the distance from the auctioneer to the destination and that for small k, there is rarely a suitable robot. If the k value is too big, the communication costs are much higher, while network lifetime improvement is almost negligible. For the BFS algorithm, different values of hopmax were set to 7 and 10. Figure 9 shows total communication costs used for the task assignment obtained in simulations averaged for 10,000 random networks.

**Figure 10:**
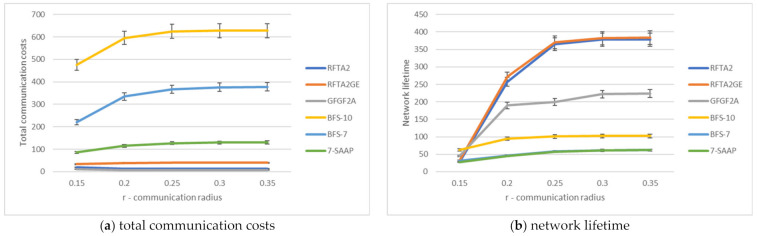
Comparison of algorithms in terms of network lifetime and communication costs.

From the simulation results presented in Figure 10, it can be seen that RFTA2 and RFTA2_GE feature a lifetime around seven times higher than 7-SAAP and BFS-7. Communication costs for 7-SAAP algorithm are up to 10 times higher than for RFTA2, and three times higher than for RFTA2GE. BFS-7 has a similar lifetime but with a higher communication overhead. BFS-10 has a lifetime twice longer than BFS-7 but with twice higher communication costs. Compared to RFTA algorithms, BFS-7 and BFS-10 have a lifetime six times smaller and communications costs that are almost 30 times higher. It can be concluded that RFTA2 and RFTA2GE outperform the k-SAAP and BFS algorithms using the combination of the face routing combined with auctions.

**Figure 11:**
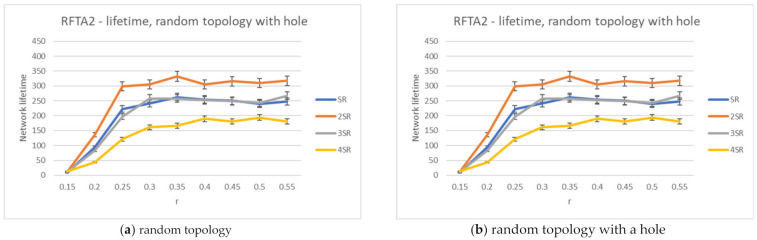
Network lifetime for the RFTA2 algorithm.

**Figure 12:**
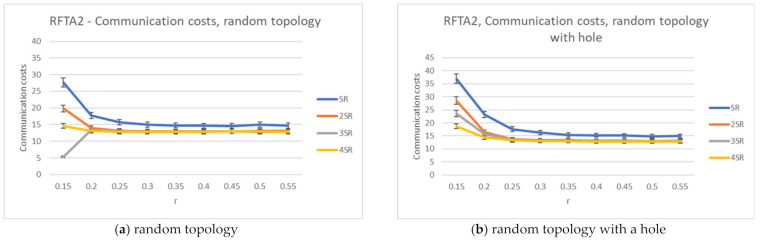
Communication costs for the RFTA2 algorithm.

Figure 11and Figure 12. We show the obtained results for proposed algorithms in detail for two different topologies and comparisons among proposed algorithms.

Figure 11 shows the average network lifetime obtained for the new RFTA2 algorithm for different radii from SR to 4SR, and communication radius r was varied from 0.15 to 0.55. It is shown that the longest average lifetime is for the 2SR, which also confirms results from Section 4.5. As the communication radius r increases, the lifetime becomes constant, starting with r > 0.20. When the communication radius is smaller, the network is mainly disconnected, and there are fewer possible routing paths, hence a shorter lifetime. For the larger r, the communication radius has no more influence on network lifetime. The longest achieved lifetime is around 350 rounds. The communication costs for different search radii from SR to 4SR and communication radius r varied from 0.15 to 0.55 are given in Figure 12. Communication costs are similar for both topologies.

**Figure 13:**
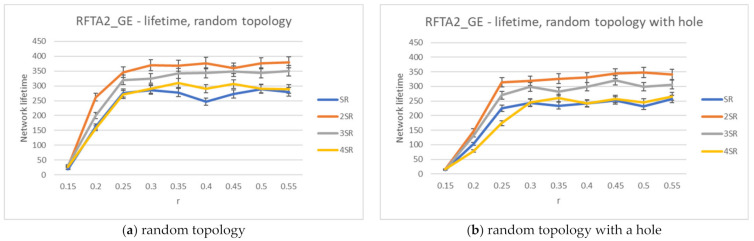
Network lifetime for the RFTA2GE algorithm for different values of SR.

**Figure 14:**
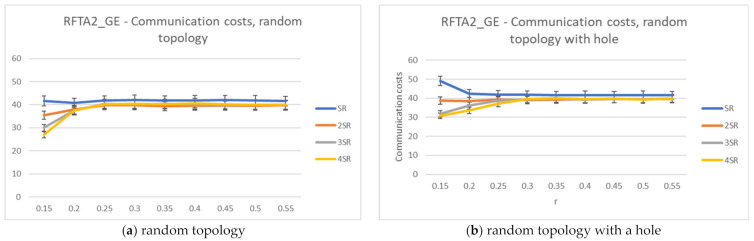
Communication costs for the RFTA2GE algorithm for different values of SR.

For the greedy extension of RFTA2 algorithm, the same set of simulations is executed, and the same performance indicators are measured. The results for the network lifetime are given in Figure 13, showing that it can take up to almost 400 rounds (a little bit lower for the topology with hole). Communication costs are given in Figure 14, showing that it is around 40 messages and similar for both topologies.

**Figure 15:**
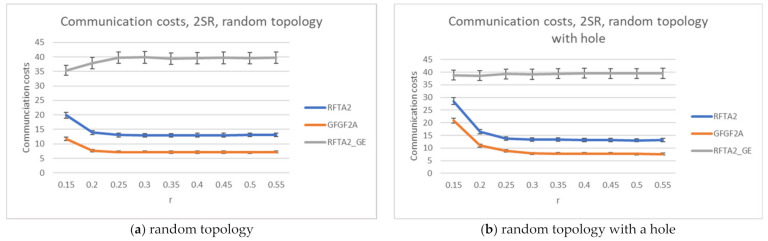
Communication costs comparison for RFTA2, GFGF2A, and RFTA2GE.

**Figure 16:**
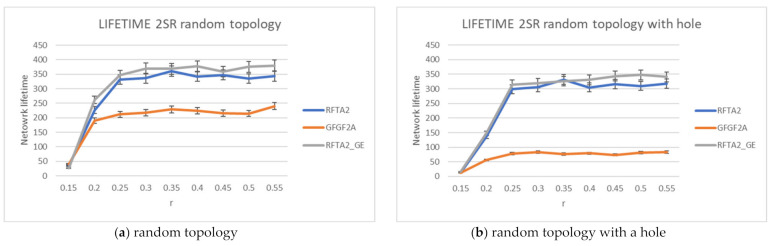
Network lifetime comparison for RFTA2, GFGF2A, and RFTA2GE.

The simulations showed that the best results are obtained for the search radius of 2SR (as concluded in [7] also), as explained in Section 4.5. Accordingly, communication costs and network lifetime are compared for the RFTA2, GFGF2A, and RFTA2GE for fixed search radius 2SR. From Figure 15, it can be seen that, as expected, the highest communication costs are for the RFTA2GE (i.e., greedy extension of RFTA2) algorithm for both topologies. It is around four times higher than the other two algorithms. On the other hand, it can be seen from Figure 16 that the network lifetime of the new algorithms RFTA2 and RFTA2GE is almost doubled compared to the GFGF2A algorithm for the random topology scenario, and it is nearly four times longer for random topology with the hole. If we compare RFTA2 and RFTA2GE, communication costs for greedy extension are much higher for minimal benefit gained in network lifetime.

**Figure 17:**
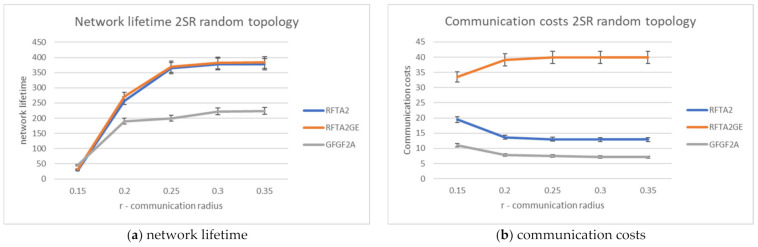
Comparisons for RFTA2, GFGF2A, and RFTA2GE for random topology.

What is shown in Figure 16, furthermore, is that the lifetime for GFGF2A for the random topology with a hole is four times shorter than for the random topology. The explanation is as follows. The network changes more in topology with hole since the hole occupies a significant part of the area while a task can be found anywhere within a unit square. Thus, a robot doing the task could be moving a lot and would therefore lose lots of energy. On the other hand, in the RFTA2 and RFTA2GE (having auctions), the robots with more energy are chosen, and hence the hole in topology does not influence the network lifetime. Figure 17 shows the same simulation results but obtained for 10,000 simulations and with a smaller range for r. The results are very similar and the differences from 100 to 10,000 simulations are negligible.

**Figure 18:**
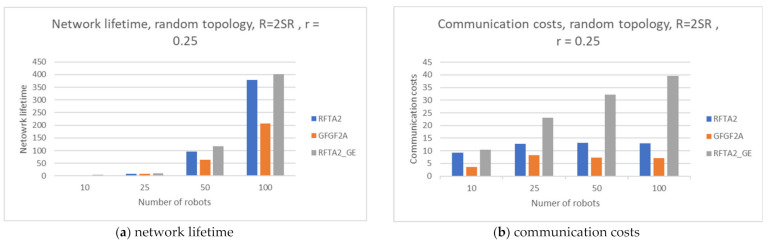
Comparisons for RFTA2, GFGF2A, and RFTA2GE for changing number of robots (10, 25, 50, and 100) for random topology and SR = 0.2, r = 0.25.

Additionally, we explored the influence of the number of robots on the overall performance. For these simulations, we used random topology, SR and r were fixed to 0.2 and 0.25, respectively, and the number of robots was 10, 25, 50, and 100. The results are presented in Figure 18.

Figure 18a shows that the network lifetime drops significantly when the number of robots decreases. The lower the number of robots, the network become less dense with fewer routing paths. On the other hand, the network lifetime depends on the robot’s energy, and when a robot performs a task, its energy decreases depending on the distance to the event. In scenarios with fewer robots, the distance to the event can be high, and the robot energy depletes very fast. That is why the network lifetime is shorter as the number of robots decreases. Communication costs increase as the number of robots increases, as expected. In scenarios with more robots, there are more routing paths. However, it also incurs higher communication costs.

### Robot Energy Statistics and Balancing

**Figure 19:**
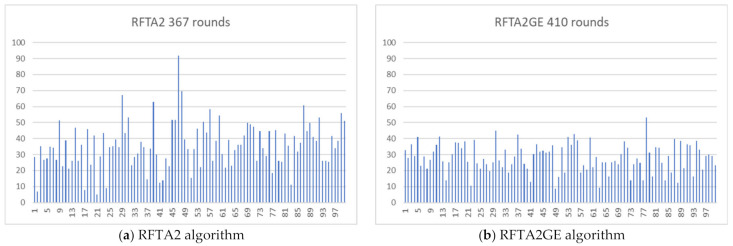
Robot energies after the lifetime at the random topology for (SR = 0.2, r = 0.25).

Another measured parameter is the energy each robot has been left with after the round in which the network is considered not alive (i.e., network lifetime is achieved). Figure 19 shows two examples, one for RFTA2 and one for RFTA2GE algorithm. In both cases, the routing started on the same network. RFTA2GE features a longer network lifetime (as expected), and the energies are also better balanced.

Figure 19 shows that there are still many robots with significant remaining energy. As the robot energy dries out, the probability that the network will be disconnected rises. However, that scenario is out of the scope of this work. There is room for further optimizations, some of which we identify in the conclusion section.

The following variables are measured with standard deviation to better explore the energy statistics and balancing among robots for all three algorithms. Average messages per robot variable measure how many messages each robot has sent during the network lifetime. The difference related to the communication costs variable measuring the number of messages sent during the *single* routing task is to be noted. Other averaged variables presented are the network lifetime, minimum of remaining robot energy, remaining robot energy, number of robot reactions, and traveled distance per robot. Table 2 and Table 3 show obtained statistics for random topology and topology with hole.

It can be concluded that the RFTA1 algorithm is not better compared to the GFGF2 algorithm in terms of finding the closest robot. Moreover, it has around 25% higher communication costs.

RFTA2 is better compared to GFGF2A in terms of longer network lifetime since it features a 1.6-times longer network lifetime with 1.8-times higher communication costs for the random topology and a 3.75-times longer lifetime with 1.8-times higher communication costs for the random topology with hole. The greedy extension of RFTA2 has little benefit on the network lifetime compared with RFTA2 (around 7%), with approximately 2.5-times higher communication costs.

For the random topologies, RFTA2GE features the best robot utilization and energy balancing of all algorithms. It features more than twice better robot utilization than GFGF2A and is 0.8 times better than RFTA2 in terms of average remaining robot energy. Better robot utilization can be seen from the values of the average number of robot reactions and traveled distance per robot. RFTA2GE is 1.7 times better than GFGF2A and 1.1 times better than RFTA2 in terms of robot reactions. It is more than three times better when compared to GFGF2A and 1.2 times better than RFTA2 in terms of traveled distance per robot.

For the random topologies with hole, the results show that both RFTA2 and RFTA2GE outperform GFGF2A in terms of all measured variables. RFTA2GE is almost 50% better than GFGF2A and 30% better than RFTA2 in terms of average remaining robot energy. When it comes to robot reactions, the RFTA2GE is more than nine times better than GFGF2A and six times better than RFTA2. The same trend continues for traveled distances per robot, whereby it shows six times better performance than GFGF2A and is 1.25 times than RFTA2.

After overall comparisons, we concluded that the RFTA2GE features the best energy balancing among robots. Accordingly, it is the best algorithm. Results show that communication costs (measured in the number of messages) are almost consistent for communication radius r in the range around 0.3 up to 0.55. For this range of radiuses, the network becomes denser with good versatile routes. If the energy consumption is used instead of the number of messages, it would probably incur higher communication costs. It remains to be future work.

## 6. Conclusions

In this paper, we proposed four new algorithms for the robot task allocation problem in wireless sensor and robot networks in scenarios with random and random with hole topologies—the RFTA1, RFTA2, GFGF2A, and RFTA2GE. We compared our algorithms with similar algorithms from the literature (k-SAAP and BFS) used for task assignment. The RFTA2 and RFTA2GE feature a lifetime of up to seven times longer with significant communication overhead reduction than k-SAAP and BFS. The RFTA1 algorithm does not show any benefit compared to the GFGF2 algorithm. The algorithms RFTA2 and RFTA2GE were compared to GFGF2A, and simulation results showed that RFTA2 is better than GFGF2A in terms of network lifetime with a bit of communication overhead (75% longer network lifetime for additional around ten messages higher overhead). That is particularly beneficial for random networks with a hole with the same communication overhead (4.5 times longer network lifetime). RFTA2GE (greedy extension of RFTA2) features good energy balancing, has the best network lifetime (up to 3.75 times compared to GFGF2A and up to 1.2 times compared to RFTA2), and shows solid robot utilization due to lower remaining robot energies (more than 50% lower compared to GFGF2A and around 30% lower compared to RFTA2GE) for the price of additional communication overhead. The last results are also confirmed by measuring the average number of robot reactions and the average traveled distance per robot. The fact that the communication costs are usually by an order of magnitude lower than robot movement costs is a highly beneficial feature of RFTA2GE.

For RFTA2 and RFTA2GE, using the geometric probability approach given by Lemma 1 and Lemma 2, we explained why the best results for network lifetime are obtained for SR = 2SR = 0.2. In Lemma 3 and 4, we showed that routing complexity is upper bounded by O(n*c), and communication complexity is upped bounded by *O(n^2^ + n),* respectively.

One of the limits of this work is that there is still a considerable amount of robot residual energy after the network lifetime. It seems that there is still room for further improvement. The bidding metrics used by Equation (1) could be variated with some other metrics for further comparisons. The following metrics might be used in that direction, m*d/E_R_ or m*d(E_R_ − m*d), where *m* is constant, *d* is distance, and *E_R_* is the robot’s remaining energy.

For future work, the robot’s energy loss calculation and speed could be varied to examine its impact on the results and whether more benefits could be found. It would be interesting to explore how to balance robots’ energy better while maintaining the network connected. It is planned for future research to examine the scenarios with disconnected networks and find out how robot relocation can maintain connectivity and prolong the network lifetime. A similar idea is given in [36]. It would also be interesting to compare the lifetime of the sensor part of the network with the robot part of the network. Since only succeeding events are considered in this paper, it would be interesting to explore the algorithms in the context of simultaneous events.

## Figures and Tables

**Table 1:** Analysis of task allocation algorithms.

Algorithm	Complexity	Mobility	Method	Simulation Env./Source Code
BFS [10]	O(diam … |E|)	yes	Breath-first search	Robot simulator Webots/not available
k-SAAP [9]	N/A	yes	Localized auctions with k neighbors	C programming language/available
AAP [24]	N/A	no	Localized auctions with an adaptive number of neighbors	OMNet++ simulation tool /not available
MIA-TA [36]	O(n^2^)	yes	Auctions with maintaining network connectivity	Python on a PC with AMD 4.1 GHz CPU and 8GB RAM/not available
RFTA2	O(n^2^ + n)	yes	GFG with auctions	C programming on Intel i5 @ 1.9 GHz, 8GB RAM

**Table 2:** Robot energies statistics after the final rounds for the random topology.

	GFGF2A	RFTA2	RFTA2GE
Average messages per robot (AMPR) [#msg]	14.79 ± 5.51	43.32 ± 12.23	68.64 ± 15.39
Average network lifetime (ANL) [#rounds]	225.11 ± 88.51	323.14 ± 122.69	376.35 ± 96.94
Average MIN of remaining robot energy [%]	7.39	11.33	5.95
Average remaining robot energy (ARRE) [%]	79.52 ± 6.98	44.81 ± 20.17	35.04 ± 13.98
The average number of robot reactions (ANRR)	2.13 ± 0.81	3.07 ± 1.14	3.57 ± 0.84
Average traveled distance per robot (ATDPR) [m]	1.06 ± 0.36	2.85 ± 1.04	3.35 ± 0.72

**Table 3:** Robot energy statistics after the final rounds for the random topology with a hole.

	GFGF2A	RFTA2	RFTA2GE
Average messages per robot (AMPR) [#msg]	6.45 ± 3.63	13.62 ± 6.29	57.62 ± 18.13
Average network lifetime (ANL) [#rounds]	85.60 ± 57.25	260.16 ± 123.88	319.87 ± 105.66
Average MIN of remaining robot energy [%]	11.49	12.00	8.03
Average remaining robot energy (ARRE) [%]	90.56 ± 5.48	53.44 ± 20.76	41.93 ± 16.88
The average number of robot reactions (ANRR)	0.82 ± 0.56	2.47 ± 1.15	3.05 ± 0.95
Average traveled distance per robot (ATDPR) [m]	0.49 ± 0.28	2.39 ± 1.07	2.99 ± 0.87
